# Social differences in COVID-19 vaccination rates–Findings from a German nationwide study

**DOI:** 10.1093/eurpub/ckac131.410

**Published:** 2022-10-25

**Authors:** S Bartig, S Müters, J Hoebel, NK Schmid-Küpke, C Hövener

**Affiliations:** 1 Epidemiology and Health Monitoring, Robert Koch Institute, Berlin, Germany; 2 Infectious Disease Epidemiology, Robert Koch Institute, Berlin, Germany

## Abstract

**Background:**

The COVID-19 vaccination aims to prevent the transmission of the novel coronavirus (SARS-CoV-2) as well as to reduce severe courses of the disease and deaths. But various studies indicate social differences in willingness to be vaccinated. This study aims to examine the influence of different social determinants on COVID-19 vaccination in Germany.

**Methods:**

The analyses are based on data from the sixth follow-up survey of the German Health Update (GEDA 2021), a nationwide cross-sectional telephone survey of the adult population living in Germany. COVID-19 vaccination rates are analyzed considering age, education, income, urban-rural residence and migration background. Poisson regressions were used to examine associations of each social determinant with COVID-19 vaccination rates. Adjustments were made for age, sex, (education) and date of participation.

**Results:**

Overall, the rate of COVID-19 vaccination for the survey period was 86.7%, with significant differences in vaccination rate by social determinants. The vaccination rate increases significantly with age (94.2% for over 60-year-olds), higher level of education (91.5%) or income (93.0%). In addition, people living in rural areas in Germany (83.5%) and people with an own migration experience (79.1%) had a significantly lower vaccination rate. An age-differentiated analysis also showed the social differences in COVID-19 vaccination rate are significantly lower among those over 60-years old.

**Conclusions:**

The results suggest social differences in COVID-19 vaccination especially in younger age groups. This should be considered when designing targeted measures to overcome potential barriers to vaccination. However, a large number of other factors affecting vaccination behavior must be taken into account like structural barriers, confidence in decision-makers, the safety of vaccination, and a sense of responsibility towards the community.

**Key messages:**

• Sociodemographic and socioeconomic determinants affect COVID-19 vaccination rates.

• The social differences in COVID-19 vaccination are lower among those over 60-years old.

